# Characteristics of cardiopulmonary exercise testing in patients with combined post- and pre-capillary pulmonary hypertension due to left heart disease

**DOI:** 10.1371/journal.pone.0286057

**Published:** 2023-05-22

**Authors:** Ayumi Goda, Yoshiaki Yanagisawa, Shinsuke Takeuchi, Kaori Takeuchi, Hanako Kikuchi, Takumi Inami, Takashi Kohno, Toru Satoh, Kyoko Soejima

**Affiliations:** Department of Cardiovascular Medicine, Kyorin University Hospital, Tokyo, Japan; University of Minnesota Medical Center Fairview: M Health Fairview University of Minnesota Medical Center East Bank, UNITED STATES

## Abstract

**Background:**

Pulmonary hypertension (PH) is a common and morbid complication of left heart disease (LHD), comprising two subtypes: (1) isolated post-capillary pulmonary hypertension (Ipc-PH) and (2) combined post-capillary and pre-capillary pulmonary hypertension (Cpc-PH). Knowledge regarding the physiological characteristics that distinguish Cpc-PH, which has a worse prognosis, from Ipc-PH remains limited. Therefore, this study aimed to assess the utility of cardiopulmonary exercise testing (CPET) variables in detecting Cpc-PH.

**Methods and results:**

Among 105 consecutive patients with LHD (age: 55 ± 13 years; male/female = 79/26) who underwent right heart catheterization and CPET, 45 (43%) were classified as PH-LHD (mean pulmonary artery pressure >20 mmHg). Ipc-PH (n = 24) was defined as pulmonary vascular resistance (PVR) ≤ 3 WU and Cpc-PH (n = 21) as PVR > 3 WU. Patients with Cpc-PH had a significantly lower peak partial pressure of carbon dioxide (PETCO_2_) (Non-PH/Ipc-PH/Cpc-PH = 38.2 ± 6.6 vs. 38.3 ± 6.0 vs 33.0 ± 4.4 mmHg, p = 0.006), higher VE vs. VCO_2_ slope (Non-PH/Ipc-PH/Cpc-PH = 33.0 [28.3, 36.6] vs. 32.5 [28.1, 37.8] vs. 40.6 [33.6, 46.1], p = 0.007), and lower ΔVO_2_/ΔWR (Non-PH/Ipc-PH/Cpc-PH = 8.5 ± 1.4 vs. 8.0 ± 1.7 vs. 6.8 ± 2.0 mL/min/watt, p = 0.001) than those with Ipc-PH and non-PH. Using multivariable logistic regression analysis, CPET variables were found to be independent predictors of Cpc-PH (lower peak PETCO_2_: odds ratio, 0.728 [95% confidence interval {CI}: 0.616–0.840], p = 0.003 and lower ΔVO_2_/ΔWR: odds ratio, 0.747 [95% CI: 0.575–0.872], p = 0.003).

**Conclusion:**

From our exploratory analysis, CPET variables, especially in the lower peak PETCO_2_ and lower ΔVO_2_/ΔWR, were associated with Cpc-PH in patients with left heart disease.

## Introduction

Pulmonary hypertension (PH) is a common and morbid complication of left heart disease (LHD). It comprises the following two subtypes: (1) isolated post-capillary PH (Ipc-PH) and (2) combined post-capillary and pre-capillary PH (Cpc-PH) [1]. Pathologically and genetically, Cpc-PH is considered to possess characteristics that are intermediate between those of pulmonary arterial hypertension (PAH), that is pre-capillary PH, and LHD, that is post-capillary PH [2–4]; furthermore, it has a worse prognosis than that of Ipc-PH. However, knowledge regarding the clinical or physiological characteristics that distinguish between these two sub-phenotypes remains limited [5]. The efficacy of pulmonary vasodilators in Cpc-PH has been explored recently [6, 7]. Despite the increased importance of detection and differentiation of Cpc-PH to enable a tailored approach for PH treatment, an invasive evaluation for PH remains mandatory.

The cardiopulmonary exercise test (CPET) is a well-established noninvasive test for assessing functional capacity and exercise limitation. Based on pathophysiology, it provides mechanistic insights and important information on gas exchange, ventilatory efficacy, and cardiac function during exercise [8, 9]. Peak oxygen consumption (peak VO_2_), which reflects the cardiac output (CO) during exercise, and other ventilatory variables (such as the ventilation/carbon dioxide-production relationship slope [VE vs. VCO_2_ slope]) remain the most frequently applied variables in the CPET and have been used as markers of disease severity and prognosis in patients with heart failure (HF) and PAH [10–13]. In terms of the CPET, PAH is characterized by a lower peak VO_2_, marked hyperventilation, a low end-tidal partial pressure of carbon dioxide (PETCO_2_), and an elevated VE vs. VCO_2_ slope [13, 14]. The combination of a low PETCO_2_ and high ventilatory equivalents for carbon dioxide (VE/VCO_2_) at the anaerobic threshold (AT) has been considered suggestive of PH [15]. Previous reports on the differentiation of Cpc-PH have proposed the usefulness of various ventilatory parameters, such as the VE/VCO_2_ at AT, PETCO_2_, dead-space ventilation-to-tidal ventilation (VD/VT), and lowest VE/VCO_2_%pred; however, no consensus has been reached yet [16, 17].

An examination of the CPET variables in patients with Cpc-PH may provide mechanistic insights into the pathophysiology of Cpc-PH and facilitate the detection of early abnormal adaptations leading to Cpc-PH. Therefore, this study aimed to assess the utility of CPET variables in the non-invasive detection of Cpc-PH, which is considered to possess characteristics of both pre-capillary and post-capillary components.

## Methods

This study complied with the principles of the Declaration of Helsinki and was approved by the Committee for Clinical Studies and Ethics of the Kyorin University School of Medicine (No 1261). Written informed consent was waived by the ethics committee because of the retrospective nature of this study.

### Study patients

We retrospectively enrolled patients with LHD undergoing both right heart catheterization (RHC) and CPET under stable clinical conditions at our hospital between 2012 and 2017. During this period, elective RHC was performed in 255 patients whose primary diagnosis was LHD. Patients whose primary diagnosis was not LHD (e.g., right-sided HF, constrictive pericarditis, high-output HF) and those whose RHC was performed in urgent settings (e.g., acute coronary syndrome, cardiogenic shock) were not included. The clinical indication for RHC was according to the international guideline [18]. In total, 2,211 patients, without comorbidities influencing exercise performance or usual contraindications for CPET, underwent CPET for the purpose of exercise prescription or exercise tolerance assessment. Finally, the study population comprised 105 consecutive hospitalizedpatients with LHD who underwent both RHC and CPET. Patients with dilated cardiomyopathy (n = 56), hypertrophic cardiomyopathy (n = 3), secondary cardiomyopathy (n = 6), ischemic cardiomyopathy (n = 13), valvular heart disease (n = 13), hypertensive heart disease (n = 5), and diastolic dysfunction (n = 9) were included.

### Right heart catheterization

RHC was performed using a 6-F double-lumen, balloon-tipped, flow-directed Swan–Ganz catheter (Harmac Medical Products, Inc., Buffalo, NY, USA) via the transjugular approach.

The baseline hemodynamic data were recorded; the zero-reference level (mid-chest) was adjusted at the commencement of pressure measurement, and the pulmonary artery wedge pressure (PAWP) was obtained as the mean value of the arterial trace during occlusion. Measurements were obtained at the end of a normal expiration with the patients in a resting-state supine position to assess the right chamber, right atrium pressure (RAP), right ventricular end-diastolic pressure, pulmonary artery pressure (PAP; mean PAP, systolic PAP, and diastolic PAP), and PAWP [19]. The O_2_ saturation in the pulmonary artery (SvO_2_) and in the arterial blood i.e. in the radial or femoral artery (SaO_2_) was measured. The CO was determined by the Fick method using the following formula: CO (L/min) = VO_2_/(1.34 × hemoglobin × [SaO_2_ –SvO_2_]). The pulmonary vascular resistance (PVR) was calculated as follows: PVR (Wood units [WU]) = (mean PAP–PAWP)/CO. The diastolic pulmonary pressure gradient (DPG) was calculated as follows: DPG = diastolic PAP–PAWP. The pulmonary arterial compliance (PAC) were calculated as: PAC (ml/mmHg) = stroke volume/ pulse pressure.

### Hemodynamic definition

To investigate the hemodynamics according to the presence of pulmonary vasculopathy, we divided patients into the following PH subgroups according to the recommendations in 2019 [1]: (i) non-PH group (mean PAP ≤ 20 mmHg), (ii) Ipc-PH group (mean PAP >20 mmHg with PVR ≤ 3.0 WU), and (iii) Cpc-PH group (mean PAP >20 mmHg with PVR >3.0 WU).

### CPET

An incremental symptom-limited exercise test was performed within 3 weeks of RHC using an electromagnetically braked cycle ergometer (Strength Ergo 8, Fukuda Denshi, Tokyo, Japan) according to the ramp protocol. The test comprised a 3-minute resting period, followed by 3 min of warm up at an ergometer setting of 10 W (60 rpm); this was subsequently followed by testing that involved a 1–2 W increase in the exercise load every 6 s (10–20 W/min), depending on the predicted maximum exercise capacity, such that a maximal effort was attained within 8–15 min. The heart rate, arterial blood pressure in the brachial artery, and electrocardiogram were monitored continuously during the test.

During exercise, oxygen consumption (VO_2_), carbon dioxide output (VCO_2_), and minute ventilation (VE) were measured using a metabolic cart (AE-302S; MINATO, Tokyo, Japan). Prior to calculating the parameters from the respiratory gas analysis, an eight-point moving average of the breath-by-breath data was obtained. Peak VO_2_ was defined as the average value obtained during the last 30 s. The AT point was determined using the *V*-slope method in addition to the following conventional criteria: VE/VO_2_ increased after registering as flat or decreasing, whereas VE/VCO_2_ remained constant or decreased [20, 21]. The VE vs. VCO_2_ slope was calculated from the commencement of incremental exercise to the respiratory compensation (RC) point using least squares linear regression [11]. The PETCO_2_ was recorded at rest, AT, and peak exercise. The slope of VO_2_ increase to work-rate increase (ΔVO_2_/ΔWR), reflecting the rate of CO increase, was calculated from the data recorded between 30 s after the commencement of incremental exercise and 30 s before the end of the exercise using least squares linear regression.

### Echocardiography

Transthoracic Doppler echocardiography was performed, and echocardiograms were stored digitally on an ATRADA (Cannon, Japan) ultrasound system. The frame rate was maintained at a minimum of 60/s. For Doppler recordings, an average of 3–5 consecutive beats were measured using a horizontal sweep of 75–100 cm/s.

The left ventricular (LV) dimensions and left atrial diameter (LAD) were measured from the parasternal long axis view. The LV ejection fraction (LVEF) was calculated using Simpson’s biplane method from the apical four- and two-chamber views. Mitral inflow was assessed in the apical four-chamber view with the pulsed-wave Doppler sample volume placed at the tips of the mitral valve leaflets during diastole; the early (E) and late (A) peak diastolic velocities of mitral inflow and the E wave deceleration time were thus measured. Mitral annular motion was assessed using pulsed-wave tissue Doppler with the sample volume placed in the septal (e’ septal). The E/e ratio was calculated.

### Statistical analysis

Analyses were performed using SPSS (version 26.0; IBM Corp., Armonk, NY, USA). The Shapiro–Wilk test and histogram analyses were performed to assess for normality. Continuous variables are presented as mean ± standard deviation or median (25^th^, 75^th^ interquartile range), where appropriate. Comparisons of more than two groups were performed using a one-way analysis of variance (with the Bonferroni post-hoc test) or the Kruskal–Wallis test (with the Dunn’s post-hoc test), where appropriate. Categorical variables are presented as percentages and were compared using the Fisher’s exact test or the Pearson’s χ^2^ test. Univariable and multivariable logistic regression analyses were performed to predict Cpc-PH using the CPET parameters. Significant independent variables were identified using stepwise selection (p<0.05). Receiver operating characteristic curves were constructed, and the area under the curve (AUC) was calculated. The cutoff value resulting in the highest product of sensitivity and specificity was considered optimal for the detection of Cpc-PH. The relationship between hemodynamic phenotype and all-cause death was evaluated with Kaplan–Meier analysis. Differences between survival curves were assessed using the log-rank test. The association between the CPET parameters and mortality was assessed using Cox proportional hazard models. Variables that were significant in the univariate analysis were entered into the multivariate Cox proportional hazard models. Statistical significance was set at p <0.05.

## Results

### General characteristics

**Fig 1:**
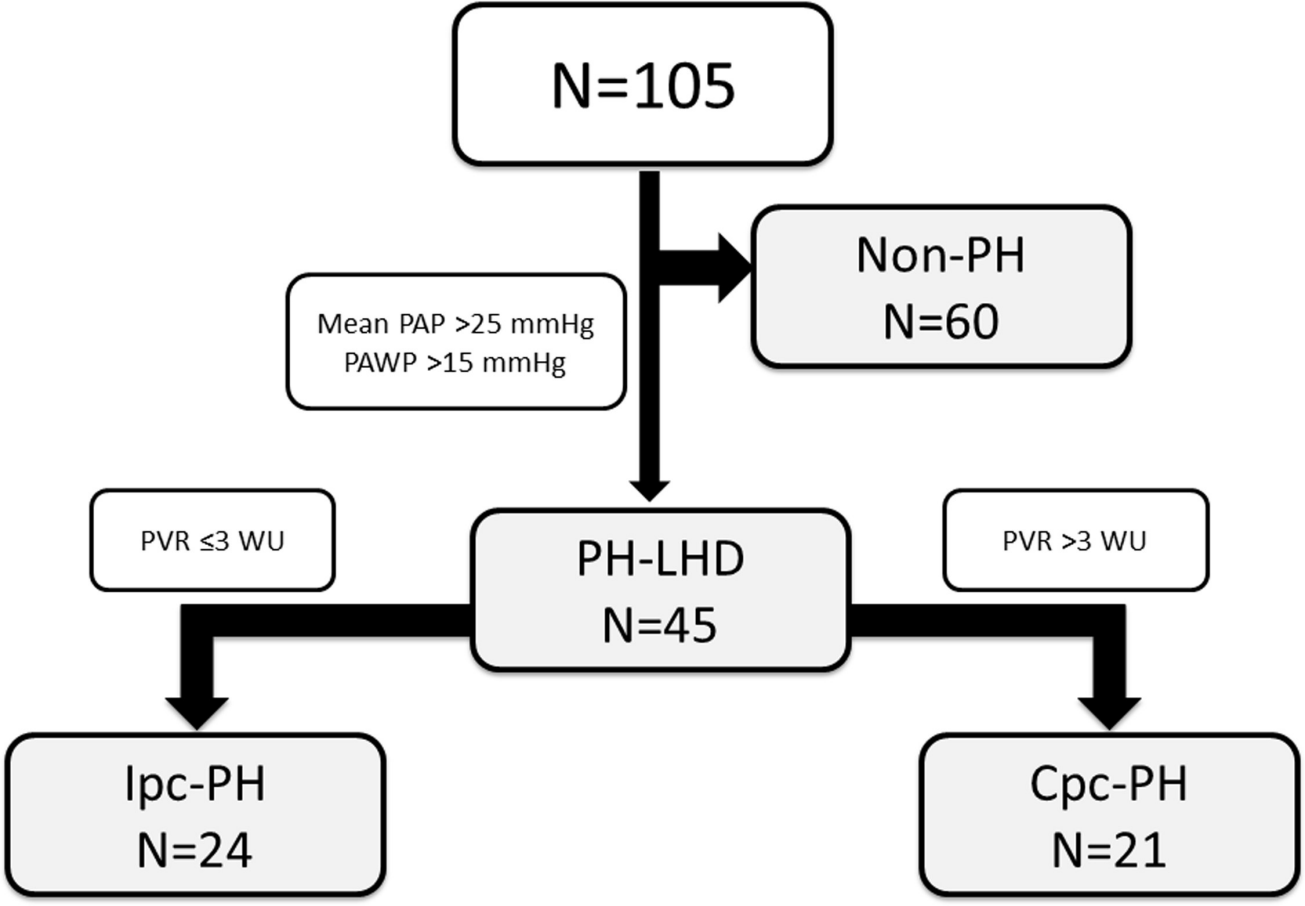
Flow chart of patients with LHD who underwent the CPET and RHC. Cpc: combined post-capillary and pre-capillary, CPET: cardiopulmonary exercise test, Ipc: isolated post-capillary, LHD: left heart disease, PAP: pulmonary artery pressure, PAWP: pulmonary artery wedge pressure, PH: pulmonary hypertension, PVR: pulmonary vascular resistance, RHC: right heart catheterization.

The 105 enrolled patients with LHD who underwent RHC and the CPET were predominantly male (75%), with a mean age of 55 ± 13 years and a mean LVEF of 39 ± 14%. Overall, 45 patients (43%) were classified as having PH-LHD. Among them, 24 patients (23%) had Ipc-PH and 21 patients (20%) had Cpc-PH (Fig 1). The general characteristics of the non-PH, Ipc-PH, and Cpc-PH groups are shown in Table 1. There were no significant differences in the age, LHD etiology, and hemoglobin level among the three groups. Patients in the Cpc-PH group were predominantly male, had higher brain natriuretic peptide levels, and had lower LVEF as compared to the levels of those in the non-PH and Ipc-PH groups.

**Table 1:** Baseline characteristics of the patients.

	Non-PH (n = 60)	Ipc-PH (n = 24)	Cpc-PH (n = 21)	P value	Non-PH vs Ipc-PH	Non-PH vs Cpc-PH	Ipc-PH vs Cpc-PH
**Age, years**	55 ± 13	52 ± 10	59 ± 13	0.229	0.994	0.812	0.261
**Sex, (male/female)**	40/20	23/1	17/4	0.015			
**BMI, kg/m** ^**2**^	22.1 [20.0, 25.4]	23.4 [20.3, 27.9]	23.7 [20.8, 26.2]	0.152			
***Etiology***							
**Cardiomyopathy, n (%)**	37 (62%)	17 (71%)	11 (52%)	0.445			
**Ischemia, n (%)**	6 (10%)	2 (8%)	5 (24%)	0.201			
**Valvular, n (%)**	7 (12%)	3 (13%)	3 (14%)	0.952			
**Others, n (%)**	10 (17%)	2 (8%)	2 (10%)	0.507			
**BNP, ng/mL**	161 [68, 224]	211 [87, 445]	615 [326, 774]	<0.001	0.086	<0.001	0.005
**Hemoglobin, g/dL**	14.3 ± 2.0	14.0 ± 2.4	14.4 ± 2.3	0.726	1.000	1.000	1.000
***Echocardiography***							
**LVEF, %**	38 [29, 50]	36 [31, 38]	28 [22, 40]	0.015	1.000	1.000	1.000
**Dd, mm**	59 [53, 64]	61 [58, 66]	61 [54, 69]	0.172			
**Ds, mm**	50 [41, 55]	53 [47, 59]	54 [44, 62]	0.102			
**E/e’**	12.1 [10.3, 14.8]	15.0 [9.9, 20.6]	16.7 [11.2, 22.0]	0.058			
**LAD, mm**	40 [33, 45]	46 [41, 49]	48 [42, 55]	<0.001	0.005	<0.001	0.189
***Hemodynamic data***							
**PAWP, mmHg**	9 [7, 11]	19 [16, 22]	20 [17, 27]	<0.001	<0.001	<0.001	0.696
**Systolic PAP, mmHg**	26 [22, 31]	42 [35, 46]	49 [40, 58]	<0.001	<0.001	<0.001	0.135
**Diastolic PAP, mmHg**	11 [8, 13]	19 [16, 21]	21 [18, 29]	<0.001	<0.001	<0.001	0.296
**Mean PAP, mmHg**	16 [14, 20]	27 [23, 30]	33 [26, 41]	<0.001	<0.001	<0.001	0.132
**RVEDP, mmHg**	5 [4, 6]	8 [7, 11]	10 [8, 12]	<0.001	0.001	<0.001	0.313
**Mean RAP, mmHg**	4 [3, 6]	7 [5, 9]	9 [6, 12]	<0.001	<0.001	<0.001	0.187
**DPG, mmHg**	1 [-1, 3]	0 [-3, 2]	1 [-1, 4]	0.066			
**SaO**_**2**_ **, %**	97 [95, 98]	97 [96, 98]	96 [94, 98]	0.524			
**SvO**_**2**_ **, %**	71 [69, 74]	70 [66, 73]	64 [57, 68]	<0.001	0.252	<0.001	0.004
**CO, L/min**	3.8 [3.1, 4.6]	4.0 [3.3, 5.0]	3.2 [2.6, 3.8]	0.004	0.363	0.005	0.002
**PVR, Wood Units**	1.7 [1.2, 2.4]	2.0 [1.1, 2.2]	3.2 [3.0, 4.4]	<0.001	0.756	<0.001	<0.001
**PAC, ml/mmHg**	3.2 [2.5, 4.7]	2.4 [2.0, 3.2]	1.6 [1.1, 2.0]	<0.001	0.018	<0.001	0.005

Values are reported as the mean ± SD or median (25th, 75th interquartile range), where appropriate.

BMI: body mass index, BNP: brain natriuretic peptide, CO: cardiac output, Cpc: combined post-capillary and pre-capillary, Dd: Dimension diastolic, DPG: diastolic pressure gradient, Ds: Dimension systolic, Ipc: isolated post-capillary, LAD: left atrial diameter, LVEF: left ventricular ejection fraction, PAC: pulmonary arterial compliance, PAP: pulmonary artery pressure, PAWP: pulmonary artery wedge pressure, PH: pulmonary hypertension, PVR: pulmonary vascular resistance, RAP: right atrium pressure, RVEDP: right ventricular end-diastolic pressure, SaO_2_: arterial oxygen saturation, SvO_2_: mixed venous oxygen saturation.

### Hemodynamic parameters

The hemodynamic parameters of the study groups are shown in Table 1. The PAWP, PAP, and mean RAP were significantly higher in the Ipc-PH and Cpc-PH groups than in the non-PH group. However, these variables did not differ between the Ipc-PH and Cpc-PH groups. The CO was significantly lower in the Cpc-PH group than in the non-PH and Ipc-PH groups (non-PH vs. Ipc-PH vs. Cpc-PH = 3.8 [3.1, 4.6] vs. 4.0 [3.3, 5.0] vs. 3.2 [2.6, 3.8] L/min, p = 0.004). The PVR was significantly higher in the Cpc-PH group than in the non-PH and Ipc-PH groups (non-PH vs. Ipc-PH vs. Cpc-PH = 1.7 [1.2, 2.4] vs. 2.0 [1.1, 2.2] vs. 3.2 [3.0, 4.4] WU, p<0.001).

### CPET parameters

**Fig 2:**
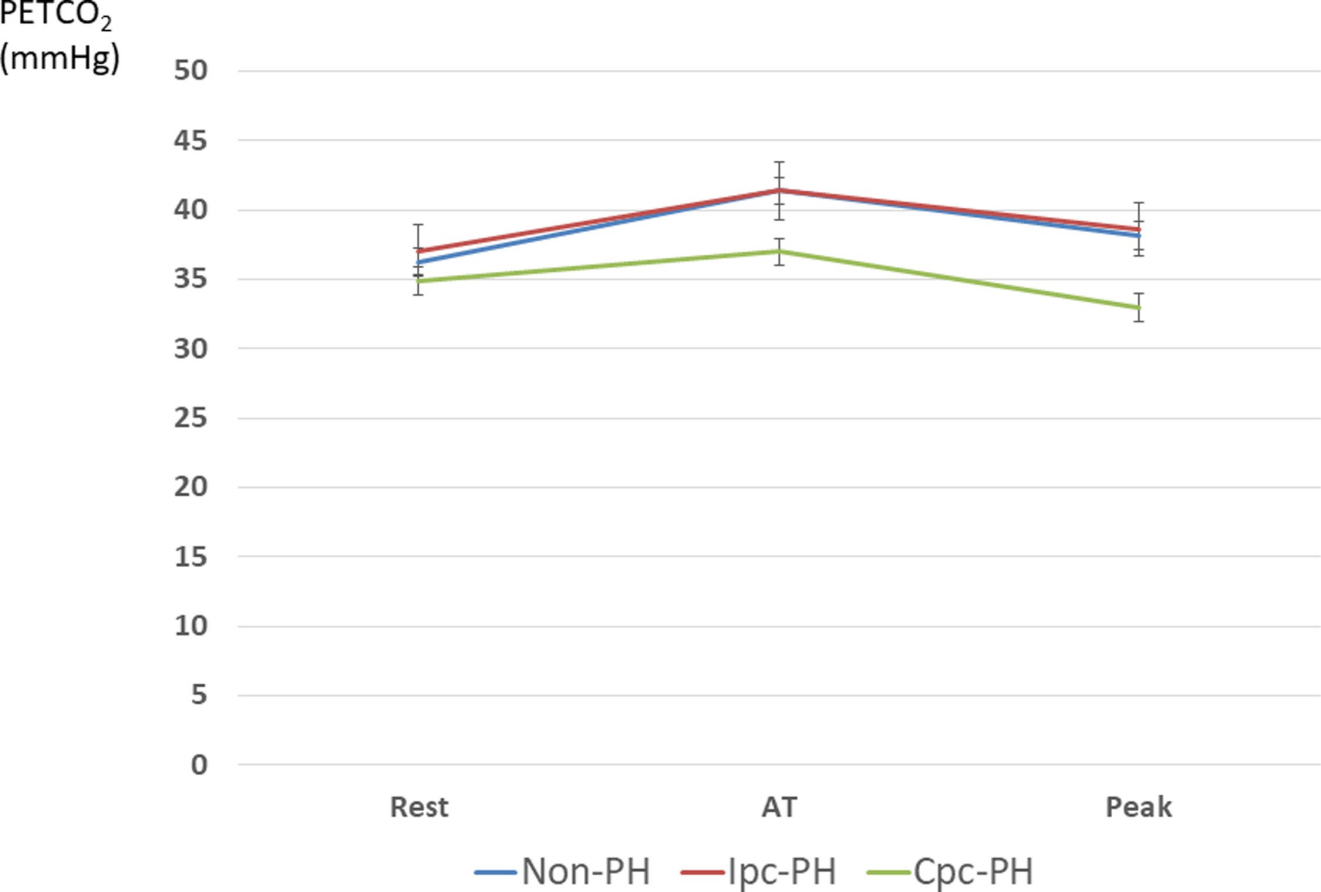
Evolution of the PETCO_2_ from rest to peak exercise in the three groups of patients. AT: anaerobic threshold, Cpc: combined post-capillary and pre-capillary, Ipc: isolated post-capillary, PETCO_2_: end-tidal partial pressure of carbon dioxide, PH: pulmonary hypertension.

The characteristics of the CPET variables according to the hemodynamics are listed in Table 2. Compared to the Ipc-PH and non-PH groups, the Cpc-PH group had a significantly lower peak PETCO_2_ (non-PH vs. Ipc-PH vs. Cpc-PH = 38.2 ± 6.6 vs. 38.3 ± 6.0 vs. 33.0 ± 4.4 mmHg, p = 0.002), higher VE vs. VCO_2_ slope (non-PH vs. Ipc-PH vs. Cpc-PH = 33.0 [28.3, 36.6] vs. 32.5 [28.1, 37.8] vs. 40.6 [33.6, 46.1], p = 0.007), and lower ΔVO_2_/ΔWR (non-PH vs. Ipc-PH vs. Cpc-PH groups = 8.5 ± 1.4 vs. 8.0 ± 1.7 vs. 6.8 ± 2.0 mL/min/watt; p = 0.001) (Fig 2).

**Table 2:** Exercise parameters.

	Non-PH (n = 60)	Ipc-PH (n = 24)	Cpc-PH (n = 21)	P value	Non-PH vs Ipc-PH	Non-PH vs Cpc-PH	Ipc-PH vs Cpc-PH
***Rest***							
**HR, bpm**	76 [67, 87]	80 [70, 93]	70 [68, 81]	0.148			
**VO**_**2**_**, mL/min**	222 ± 43	243 ± 43	234 ± 51	0.129	0.161	0.798	1.000
**VCO**_**2**_**, mL/min**	197 [167, 216]	213 [186, 265]	191 [170, 249]	0.128			
**R**	0.89 [0.84, 0.94]	0.89 [0.87, 0.93]	0.86 [0.82, 0.90]	0.171			
**VE, L/min**	9.8 [7.9, 10.6]	10.2 [9.0, 10.9]	10.6 [8.8, 11.4]	0.162			
**VE/VO**_**2**_	43.9 ± 7.8	43.4 ± 9.3	45.4 ± 10.0	0.705	1.000	1.000	1.000
**VE/VCO**_**2**_	48.9 ± 8.4	47.9 ± 10.0	52.3 ± 8.4	0.212	1.000	0.398	0.295
**PETCO**_**2**_**, mmHg**	36.3 ± 4.6	37.1 ± 4.3	34.9 ± 3.7	0.232	1.000	0.592	0.278
***Anaerobic threshold***							
**Work rate, Watt**	50 ± 17	49 ± 13	46 ± 21	0.706	1.000	1.000	1.000
**HR, bpm**	101 [88, 113]	93 [88, 109]	98 [89, 108]	0.533			
**VO**_**2**_**, mL/min**	700 ± 204	676 ± 160	627 ± 205	0.304	1.000	0.415	0.617
**VCO**_**2**_**, mL/min**	637 ± 193	619 ± 129	572 ± 200	0.343	1.000	0.470	0.722
**R**	0.91 [0.86, 0.94]	0.92 [0.86, 0.96]	0.92 [0.88, 0.96]	0.840			
**VE, L/min**	23.0 ± 5.0	22.8 ± 4.8	23.7 ± 6.9	0.175	1.000	1.000	1.000
**VE/VO**_**2**_	33.7 ± 6.3	33.8 ± 9.4	38.8 ± 6.4	0.023	1.000	0.023	0.077
**VE/VCO**_**2**_	37.1 ± 6.8	36.6 ± 7.6	42.8 ± 7.4	0.006	1.000	0.009	0.015
**PETCO**_**2**_**, mmHg**	41.4 ± 5.4	41.4 ± 5.5	37.0 ± 4.6	0.005	1.000	0.006	0.020
**AT VO**_**2**_**, mL/min/kg**	10.8 [9.3, 13.0]	9.5 [8.6, 11.4]	9.3 [7.5, 12.1]	0.010	0.060	0.005	0.368
***Peak***							
**Work rate, Watt**	90 ± 33	91 ± 20	85 ± 37	0.799	1.000	1.000	1.000
**HR, bpm**	128 ± 28	122 ± 29	112 ± 28	0.059	1.000	0.054	0.554
**VO**_**2**_**, mL/min**	1074 [824, 1359]	1064 [907, 1363]	916 [623, 1028]	0.099			
**VCO**_**2**_**, mL/min**	1237 ± 464	1237 ± 322	1073 ± 513	0.343	1.000	0.289	0.416
**R**	1.11 ± 0.11	1.16 ± 0.11	1.12 ± 0.12	0.320	0.396	1.000	1.000
**VE, L/min**	45.3 ± 14.4	45.2 ± 11.2	46.5 ± 18.3	0.991	1.000	1.000	1.000
**VE/VO**_**2**_	42.2 ± 8.2	43.7 ± 10.9	50.0 ± 7.3	0.002	1.000	0.001	0.014
**VE/VCO**_**2**_	37.3 [33.0, 42.6]	35.5 [30.6, 41.8]	43.4 [40.2, 51.6]	<0.001	0.385	<0.001	<0.001
**PETCO**_**2**_**, mmHg**	38.2 ± 6.6	38.3 ± 6.0	33.0 ± 4.4	0.002	1.000	0.002	0.006
**Peak VO**_**2**_ **, mL/min/kg**	17.8 ± 5.2	15.1 ± 3.1	13.8 ± 4.2	0.002	0.161	0.003	0.558
**VE vs. VCO**_**2**_ **slope**	33.0 [28.3, 36.6]	32.5 [28.1, 37.8]	40.6 [33.6, 46.1]	0.007	0.963	0.002	0.009
**ΔVO**_**2**_ **/ΔWR, mL/min/watt**	8.5 ± 1.4	8.0 ± 1.7	6.8 ± 2.0	0.001	0.727	<0.001	0.054

Values are reported as mean ± standard deviation or as median (25th, 75th interquartile range), where appropriate.

AT: anaerobic threshold, bpm: beats per minute, Cpc: combined post-capillary and pre-capillary, HR: heat rate, Ipc: isolated post-capillary, PETCO_2_: end-tidal partial pressure of carbon dioxide, PH: pulmonary hypertension, R: respiratory exchange ratio, VCO_2_: carbon dioxide output, VE: minute ventilation, VO_2_: oxygen consumption, ΔVO2/ΔWR: slope of the increase in the VO_2_ to the increase in the work rate.

### Predictors of Cpc-PH among the CPET variables

**Fig 3:**
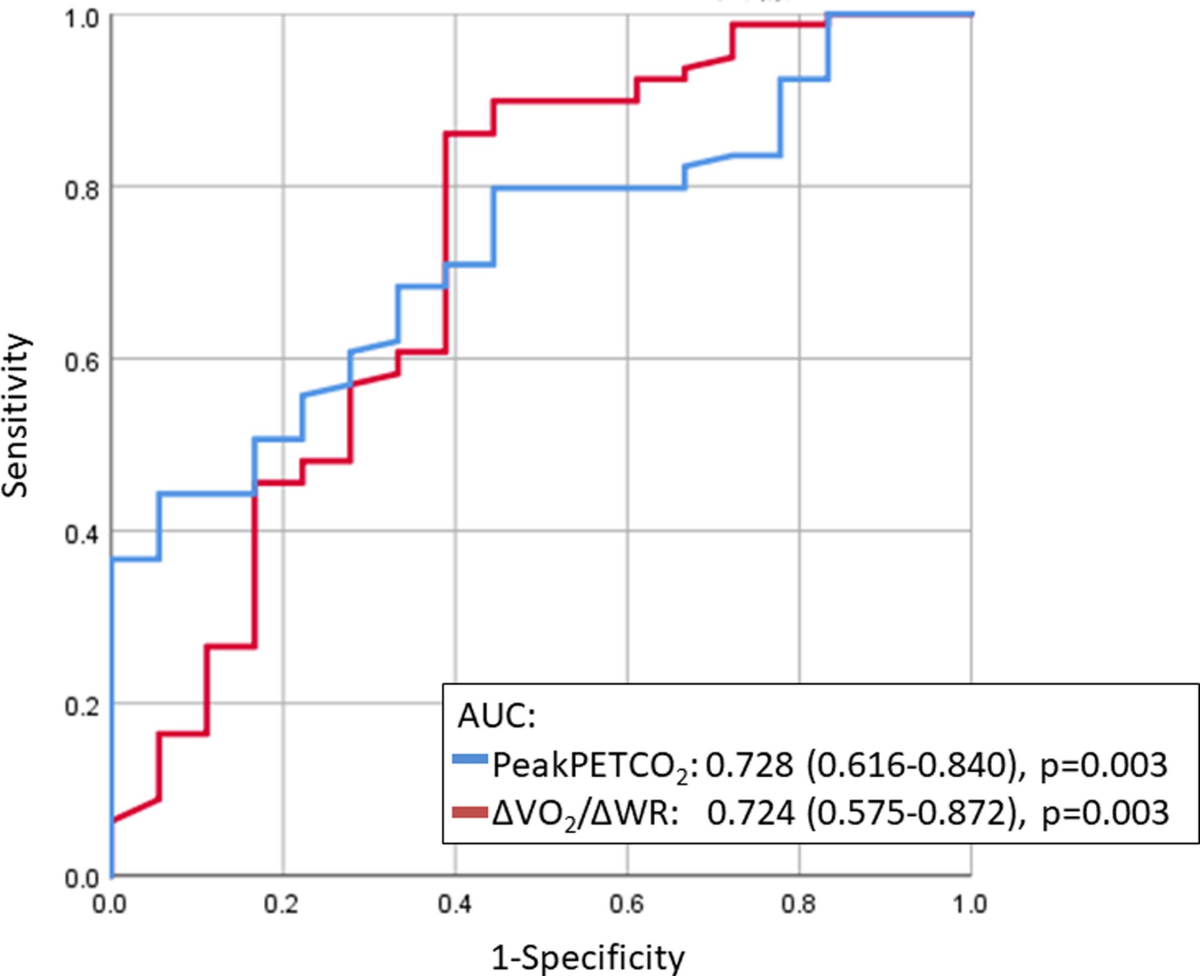
Receiver operating characteristic curves of the peak PETCO_2_ and ΔVO_2_/ΔWR to detect Cpc-PH. AUC: area under the curve, Cpc-PH: combined post-capillary and pre-capillary PH, PETCO_2_: end-tidal partial pressure of carbon dioxide, ΔVO2/ΔWR: slope of the increase in the VO_2_ to the increase in the work rate.

According to the univariable logistic regression analysis, lower peak PETCO_2_ (odds ratio, 0.838 [95% confidence interval {CI}: 0.757–0.927], p = 0.001), lower ΔVO_2_/ΔWR (odds ratio, 0.559 [95% CI: 0.395–0.792], p = 0.001), lower AT VO_2_ (odds ratio, 0.727 [95% CI: 0.558–0.945], p = 0.017), lower peak VO_2_ (odds ratio, 0.827 [95% CI: 0.724–0.945], p = 0.005), higher VE vs. VCO_2_ slope (odds ratio, 1.090 [95% CI: 1.030–1.152], p = 0.003), and higher VE/VCO_2_ at AT (odds ratio, 1.111 [95% CI: 1.036–1.191], p = 0.003) were significant predictors of Cpc-PH (Table 3). The lower peak PETCO_2_ (odds ratio, 0.868 [95% CI: 0.777–0.971], p = 0.003) and lower ΔVO_2_/ΔWR (odds ratio, 0.583 [95% CI: 0.408–0.833], p = 0.003) were the optimal predictors in the multivariable logistic regression analysis. Receiver operating characteristic curve analysis revealed that the peak PETCO_2_ (AUC: 0.728, 95% CI: 0.616–0.840, p = 0.003) and ΔVO_2_/ΔWR (AUC: 0.724, 95% CI: 0.575–0.872, p = 0.003) were indicators of Cpc-PH with predictive value (Fig 3).

**Table 3:** Univariable and multivariable logistic regression analyses for determinants of Cpc-PH.

	Univariable			Multivariable		
Variable	Odds ratio	95% CI	P value	Odds ratio	95% CI	P value
**AT VO**_**2**_	0.727	0.558–0.945	0.017			
**Peak VO**_**2**_	0.827	0.724–0.945	0.005			
**ΔVO2/ΔWR**	0.559	0.395–0.792	0.001	0.583	0.408–0.833	0.003
**VE vs. VCO**_**2**_ **slope**	1.090	1.030–1.152	0.003			
**VE/VCO**_**2**_ **at AT**	1.111	1.036–1.191	0.003			
**PETCO**_**2**_ **at rest**	0.916	0.819–1.024	0.123			
**Peak PETCO**_**2**_	0.838	0.757–0.927	0.001	0.868	0.777–0.971	0.013

CI: confidence interval, AT: anaerobic threshold, PETCO_2_: end-tidal partial pressure of carbon dioxide, VO_2_: oxygen consumption, VCO_2_: carbon dioxide output, VE: minute ventilation, ΔVO_2_/ΔWR: slope of increase in the VO_2_ to the increase in the work rate.

### Predictors of mortality among the CPET variables

**Fig 4:**
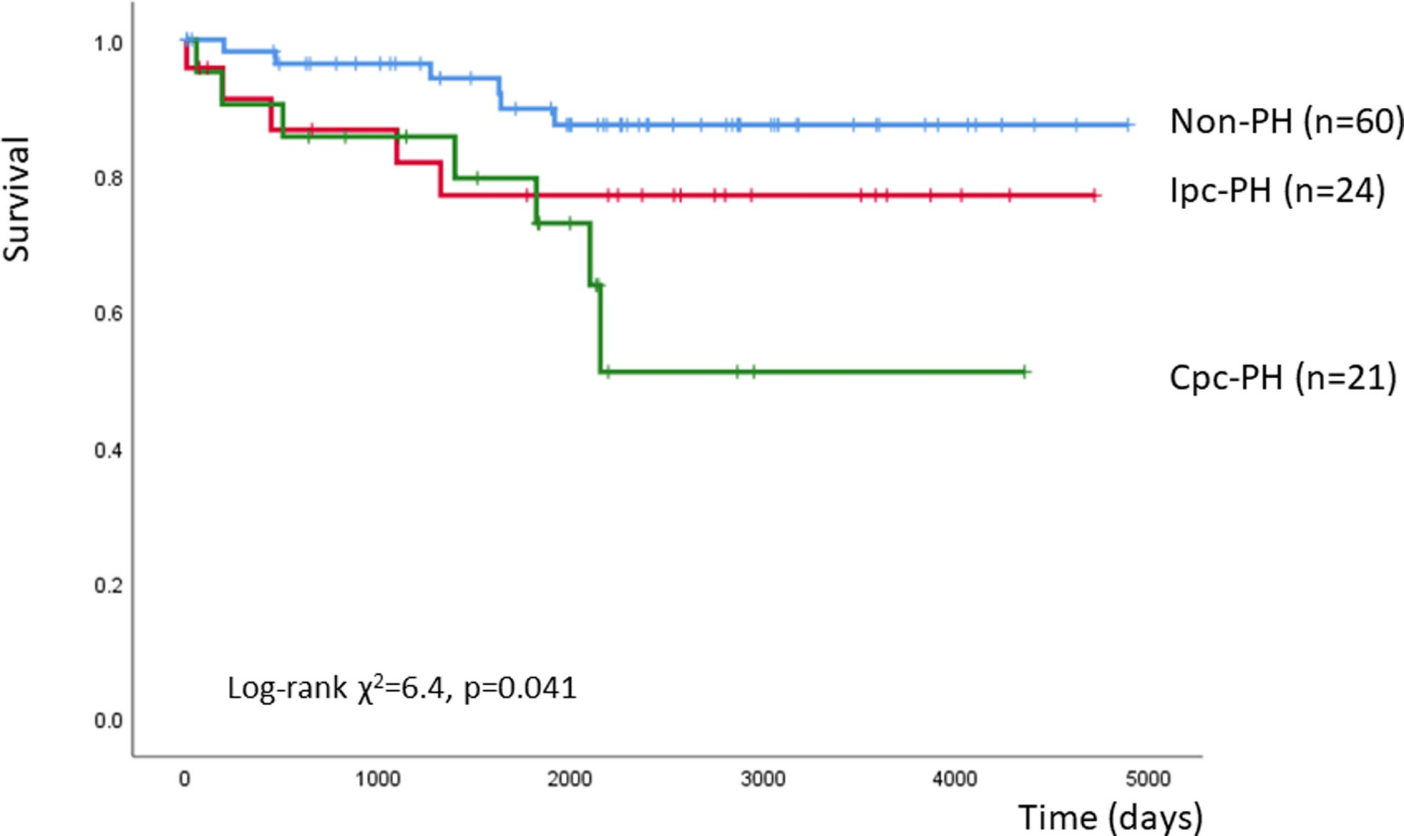
Kaplan-Meier plot stratified by PH phenotype. Cpc: combined post-capillary and pre-capillary, Ipc: isolated post-capillary, PH: pulmonary hypertension.

Over an average follow-up period of 2,108 ± 1,260 days, 18 patients died. According to the Kaplan–Meier analysis, there were significant differences in mortality among the non-PH, Ipc-PH, and Cpc-PH groups (χ^2^ = 6.4, p = 0.041) (Fig 4).

Table 4 presents the univariate predictors of death. Univariate Cox proportional hazards analysis identified lower ΔVO_2_/ΔWR (hazard ratio: 0.621, 95% CI 0.455–0.846, p = 0.003), higher VE vs. VCO2 slope (hazard ratio: 1.105; 95% CI 1.053–1.160, p<0.001), and lower peak PETCO_2_ (hazard ratio: 0.882, 95% CI 0.809–0.963, p = 0.005) as prognostic indices of death. In multivariate Cox proportional hazards analysis, lower ΔVO_2_/ΔWR (hazard ratio: 0.666, 95% CI 0.485–0.915, p = 0.012) and lower peak PETCO_2_ (hazard ratio: 0.899, 95% CI 0.813–0.994, p = 0.037) were identified as independent prognostic markers (Table 4).

**Table 4:** Univariate and multivariate Cox proportional hazard analyses for predictors of mortality.

	Univariate			Multivariate		
Variable	Hazard ratio	95% CI	P value	Hazard ratio	95% CI	P value
**AT VO**_**2**_	0.871	0.702–1.081	0.209			
**Peak VO**_**2**_	0.897	0.799–1.008	0.068			
**ΔVO**_**2**_ **/ΔWR**	0.621	0.455–0.846	0.003	0.666	0.485–0.915	0.012
**VE vs. VCO**_**2**_ **slope**	1.105	1.053–1.160	<0.001			
**VE/VCO**_**2**_ **at AT**	1.062	1.000–1.129	0.051			
**PETCO**_**2**_ **at rest**	0.875	0.897–1.096	0.875			
**Peak PETCO**_**2**_	0.882	0.809–0.963	0.005	0.899	0.813–0.994	0.037

CI: confidence interval, AT: anaerobic threshold, PETCO_2_: end-tidal partial pressure of carbon dioxide, VO_2_: oxygen consumption, VCO_2_: carbon dioxide output, VE: minute ventilation, ΔVO_2_/ΔWR: slope of increase in the VO_2_ to the increase in the work rate.

## Discussion

In our exploratory analysis, we investigated the CPET parameters in patients with non-PH, Ipc-PH, and Cpc-PH according to the criteria of 2019 [1]. In our cohort, 35% of the patients who underwent RHC had PH-LHD, with Cpc-PH accounting for 20% of the entire cohort. Therefore, Cpc-PH appeared to be a relatively uncommon condition, consistent with previous findings [17]. The present study’s results also revealed that the peak effort PETCO_2_ and ΔVO_2_/ΔWR were the optimal predictors of Cpc-PH, thereby corroborating the findings of previous studies where ventilatory variables from CPET proved useful in differentiating Cpc-PH [16, 17].

### Characteristics of Cpc-PH

PH is a common complication of LHD, and it develops in response to a passive increase in left-sided filling pressures, more specifically the left atrial pressure. While it is associated with a poor prognosis [22], Cpc-PH is known to have an even worse prognosis [5].

Genetically, Cpc-PH resembles PAH. Assad et al. found that patients with Cpc-PH had genetic abnormalities in pathways that were highly active in the lungs and were known to contribute to the pathophysiology of PAH. These exploratory genetic findings suggest that Cpc-PH may have a pathophysiology distinct from that of Ipc-PH [2].

From a pathological perspective, progressive thickening and collagen proliferation occur in the lamina densa in order to protect against fluid accumulation in the interstitium of the endothelium and the vascular wall as well as in the alveoli [3, 4, 23]. In Ipc-PH, small arteries exhibit endothelial dysfunction and vasoconstriction, despite no defined changes in the composition of small pulmonary arteries. The pulmonary veins also show a certain degree of thickness and a tendency towards arteriolarization. Moreover, in Cpc-PH, the venous system becomes fully arteriolarized, and small arteries exhibit a clear muscularization process and remodeling; an impairment of gas exchange diffusion or a lengthening of the path between air and the red blood cells is prominent.

In an effort to differentiate Cpc-PH from Ipc-PH in a non-invasive manner, a physiology-based approach is important to detect these pathological changes. In our cohort, the low value of peak effort PETCO_2_ value, which is one of the ventilatory variables in the CPET, was an optimal indicator of Cpc-PH; thus, it reflected a marked impairment of CO_2_ release into the alveolar space.

### Usefulness of peak effort PETCO_2_ in the detection of Cpc-PH

The differentiation of Cpc-PH between PAH (pre-capillary PH) and PH-LHD (post-capillary PH) using the CPET was reported by Caravita et al. [17]. They found that VE/VCO_2_ at the AT was useful in the detection of Cpc-PH and that Cpc-PH was an intermediate between PAH and Ipc-PH in terms of gas exchange. Among the ventilatory parameters obtained using the submaximal exercise test, a low PETCO_2_, high VE/VCO_2_, and high VD/VT were reportedly characteristic of Cpc-PH [16]. Moreover, the exacerbation of pulmonary gas exchange abnormalities in patients with Cpc-PH was related to an excessive rise in the pulmonary vascular pressures [16]. Zhong et al. also reported that VE/VCO_2_-related parameters were diagnostic variables for the presence of pre-capillary components in patients with PH-LHD [24]. Among the ventilatory variables, the lowest VE/VCO_2_%pred, which was obtained from the submaximal exercise test, was the optimal predictor of Cpc-PH (as demonstrated by an AUC of 0.77). From our data, the peak PETCO_2_ was also particularly useful in detecting Cpc-PH (as demonstrated by an AUC of 0.73). This is comparable to the findings reported by Zhong et al. In the maximal exercise test, the peak PETCO_2_ was the optimal diagnostic variable. Our findings (i.e., lower peak PETCO_2_ and lower ΔVO_2_/ΔWR in CpC-PH) solidified the usefulness of CPET parameters for Cpc-PH detection.

In the HF population, the PETCO_2_ is a known CPET variable that potentially possesses prognostic information [25]. In particular, Arena et al. reported that PETCO_2_ changes from rest to the RC point, PETCO_2_ at the RC point, and PETCO_2_ at peak exercise were all significant predictors of cardiac related events. Low value of PETCO_2_ during exercise have been classically considered to strongly reflect a low CO during exercise. Matsumoto et al. found that PETCO_2_ at the RC point was significantly correlated with the CO at peak exercise in patients with cardiac disease [26]. Furthermore, they concluded that the decrease in CO lead to a decrease in alveolar capillary perfusion and PCO_2_ into the alveolar air, resulting in a decrease in PETCO_2_ during exercise. Moreover, Tanabe et al. revealed a significant correlation between the PETCO_2_ and cardiac index at peak exercise in patients with HF [27].

### Mechanisms by which peak effort PETCO_2_ predicts Cpc-PH

In patients with PAH, the PETCO_2_ decline associated with exercise was more distinct than that in those with LHD [28]. Hemnes et al. demonstrated that the measurement of resting PETCO_2_ at bedside may distinguish patients with PAH from those with pulmonary venous hypertension or no PH [29]. Moreover, Welch et al. also demonstrated that this readily available resting PETCO_2_ may be a physiologically relevant marker of poor prognosis in PAH [30]. They reported that lung diffusion for carbon monoxide (DLCO) is correlated with the resting PETCO_2_, suggesting that these variables could provide potentially similar insights into the degree of pulmonary vasculopathy in patients with PAH. DLCO measures the ability of a gas to diffuse from the alveoli to the red blood cells in the pulmonary capillaries and depends on the alveolar–capillary membrane diffusive capacity and the capillary volume (which is the amount of blood flowing through the ventilated alveolar–capillary units over a period of time i.e. a few seconds) [31]. Lack of capillary perfusion leads to decrease exchanging gas into the alveolar capillaries and causes increased dead space. The correlation between the PETCO_2_ and DLCO suggests that both are markers of dead-space ventilation and that the PETCO_2_ may also reflect the capillary membrane diffusive capacity and capillary volume i.e. the CO. The peak PETCO_2_ may better capture the pathological changes in Cpc-PH, whereby the venous system becomes fully arteriolarized and gas exchange is strongly impaired.

### Usefulness of ΔVO_2_/ΔWR in the detection of Cpc-PH

The lower ΔVO_2_/ΔWR represents a poor increase in CO during exercise (a prognostic predictor), and has been suggested to be related to the severity of HF and pulmonary vascular disease in patients with HF [9, 32, 33]. Bandera et al. reported that ΔVO_2_/ΔWR flattening was an indicator of an abnormal pulmonary vascular response to exercise (i.e., right ventricular-pulmonary artery uncoupling) [34]. Consistent with these physiological findings, ΔVO_2_/ΔWR was also predictive of Cpc-PH in the present study; a lower ΔVO_2_/ΔWR may represent both a poor increase in CO during exercise and exacerbation of the pulmonary vascular dysfunction in Cpc-PH.

### Limitations

This study has certain limitations. First, our study population only included patients who were able to undergo the exercise stress test. Second, owing to the limited sample size, the interpretation of the multivariate analysis results was limited and the statistical tests were underpowered in terms of establishing novel models including several variables to predict Cpc-PH, and further studies with large study populations are warranted. Finally, our cohort comprised a heterogeneous population of patients with cardiac disease.

## Conclusions

Our exploratory study reveals that the CPET variables, especially in the lower peak PETCO_2_ and lower ΔVO_2_/ΔWR, are useful in differentiating Cpc-PH in patients with LHD. These findings might be explained, at least in part, by the presence and extent of pathologic and physiologic pulmonary vascular changes.
